# Do changes in beliefs and behaviours moderate improvement in insomnia after acquired brain injury?

**DOI:** 10.1111/jsr.14221

**Published:** 2024-05-12

**Authors:** Marthe E. Ford, Frank Verkaik, Samantha Bouwmeester, Gert J. Geurtsen

**Affiliations:** ^1^ Research and Development, Heliomare Rehabilitation Wijk aan Zee The Netherlands; ^2^ Department of Psychology, Brain and Cognition University of Amsterdam Amsterdam The Netherlands; ^3^ Tilburg School of Social and Behavioral Science Tilburg University Tilburg The Netherlands; ^4^ Department of Medical Psychology, Amsterdam UMC, Amsterdam Neurodegeneration University of Amsterdam Amsterdam The Netherlands

**Keywords:** dysfunctional beliefs and attitudes about sleep scale, insomnia severity index, sleep‐related behaviours questionnaire, stroke, traumatic brain injury

## Abstract

Key mechanisms of change in cognitive behavioural therapy for insomnia in the general population encompass changing sleep‐related beliefs and behaviours. In a population with acquired brain injury, cognitive behavioural therapy for insomnia is effective as well, but little is known about the mechanisms of change. The aim of this study was to evaluate how changing sleep‐related beliefs and behaviours were associated with improvement in insomnia following blended cognitive behavioural therapy for insomnia in a population with acquired brain injury. A secondary analysis was performed on data of a randomized–controlled trial, including 24 participants that received blended cognitive behavioural therapy for insomnia, and 24 participants that received treatment as usual. Results showed that following blended cognitive behavioural therapy for insomnia, significantly more participants improved on dysfunctional beliefs and sleep‐related behaviours and this was associated to improvement in insomnia severity. For sleep‐related behaviours, the association between improvement on behaviour and improvement on insomnia was significantly moderated by blended cognitive behavioural therapy for insomnia. However, the relation between dysfunctional beliefs and insomnia was not moderated by type of treatment. Similar results were found for acquired brain injury‐adapted versions of the questionnaires in which up to half of the items were excluded as they could be regarded as not dysfunctional for people with acquired brain injury. These results show that improvement on insomnia severity is related to improvement in dysfunctional beliefs and behaviours, and cognitive behavioural therapy for insomnia efficacy may be moderated by the improvement in behaviours in particular. A focus on these behaviours can enhance treatment efficacy, but caution is needed regarding the behaviours that may reflect adequate coping with the consequences of the acquired brain injury.

## 1 INTRODUCTION

Insomnia is the second most prevalent mental disorder in Europe (Wittchen et al., 2011), with an estimated prevalence of 10% in the general adult population (Riemann et al., 2023). In people with an acquired brain injury (ABI), insomnia prevalence is three–four times higher (Baylan et al., 2020; Mathias & Alvaro, 2012). ABI has two major causes, namely traumatic brain injury (TBI) and stroke. The global incidence of TBI is 69 million people yearly (Dewan et al., 2018). In 2019 there were 101 million stroke survivors living worldwide (Feigin et al., 2022), and this is expected to increase in the coming decades as a consequence of aging and improved survival rates (Wafa et al., 2020). Insomnia following ABI is associated with more severe physical disabilities, neuropsychiatric disturbances and cognitive impairments (Bassetti & Hermann, 2011; Baylan et al., 2020; Cantor et al., 2012; Leppavuori et al., 2002). Sleep problems can have a major effect on recovery and functional outcomes of ABI (Duss et al., 2017; Ouellet et al., 2015). Furthermore, insomnia may increase the risk of new brain injuries due to its effects on the cardiovascular system and the increased risk for accidents (Daley et al., 2009). Insomnia following ABI has often been interpreted as the consequence of pathophysiological processes linked to brain damage and specific brain circuits, psychological factors (including beliefs and behaviours), and factors linked to the environment (Ouellet et al., 2015). The high prevalence and many adverse outcomes of insomnia following ABI highlights the substantiality of the problem and the need for effective treatment.

Cognitive behavioural therapy for insomnia (CBT‐I) has shown efficacy in numerous controlled trials and is recommended as the treatment of choice for adults with insomnia, including patients with comorbidities (Riemann et al., 2023). Online CBT‐I has demonstrated similar efficacy as face‐to‐face CBT‐I (Zachariae et al., 2016). There is a growing body of evidence that CBT‐I is also effective in the ABI population (Pilon et al., 2023; Ymer et al., 2021), also when administered in a blended format combining online with face‐to‐face sessions (Ford et al., 2022). A better understanding of insomnia and CBT‐I specifically in people with ABI is highly relevant for enhancing treatment efficacy.

Sleep‐related beliefs and behaviours are considered key mechanisms of insomnia in the otherwise healthy population, and are found to mediate the outcome of both CBT‐I (Parsons et al., 2021; Sunnhed & Jansson‐Fröjmark, 2015) and online CBT‐I (Lancee et al., 2015). Ouellet et al. (2015) proposed a model of factors that contribute to sleep disturbances following TBI, and included dysfunctional beliefs (attitudes) and sleep behaviours as relevant pre‐injury and post‐acute factors, in addition to the factors more directly linked to the brain damage. Addressing these dysfunctional beliefs and behaviours may also result in better treatment outcomes for people with insomnia following ABI. However, studies on the role of sleep‐related beliefs and behaviours in insomnia following ABI are scarce. In our previous study we found that sleep‐related beliefs and behaviours are related to insomnia severity in people with ABI (Verkaik et al., 2023). Experts however rated a substantial part of the items of the questionnaires used to assess dysfunctional beliefs and behaviours as not‐dysfunctional for people with ABI (Verkaik et al., 2023). Addressing these possibly adequate coping behaviours may have unwanted effects.

To our knowledge, no previous studies have looked into whether changes in sleep‐related beliefs and behaviours are associated to post‐treatment insomnia improvement in the ABI population. In the few studies into the efficacy of CBT‐I in a population with ABI, questionnaires on sleep‐related beliefs and behaviours are mostly not included (Nguyen, McKay, et al., 2017; Nguyen, Wong, et al., 2017; Ymer et al., 2021). Pilon et al. (2023) showed an improvement of both insomnia and the sleep‐related dysfunctional beliefs following CBT‐I in an ABI population, but did not evaluate how these were related (Pilon et al., 2023). A better understanding whether the improvement of beliefs and behaviours are associated with improvement in insomnia is relevant to understand and optimize treatment outcome. In the current study, we therefore aim to: (1) replicate the finding that CBT‐I is effective in improving dysfunctional beliefs, also when administered blended (bCBT‐I), and to evaluate the effect of bCBT‐I on sleep‐related behaviours in an ABI population; (2) to evaluate whether bCBT‐I treatment effect on insomnia in an ABI population is associated with changes in sleep‐related beliefs and behaviours; (3) to evaluate if this association between bCBT‐I treatment effect and sleep‐related beliefs and behaviours is stronger when ABI‐appropriate questionnaires are used, excluding the items that may reflect non‐dysfunctional beliefs and behaviours after brain injury; and (4) to explore if the association between bCBT‐I treatment effect and sleep‐related beliefs and behaviours is weaker when related to the items that may reflect not‐dysfunctional beliefs and behaviours in the ABI population.

## 2 METHODS

### 2.1 Study design

A secondary analysis was performed on the data of a randomized–controlled trial (RCT) involving patients with insomnia following ABI, which found bCBT‐I to be an effective treatment for insomnia following ABI when compared with treatment as usual (TAU) (Ford et al., 2022). The RCT was approved by the Medical Ethical Committee of the Amsterdam University Medical Center (protocol 2017‐223) and preregistered (Trial, NL6895; NTR7082).

**FIGURE 1:**
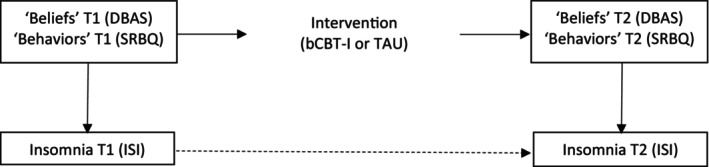
Schematic model of moderation hypotheses. Solid lines reflect relations tested in the model. Dashed line reflects possible changes in insomnia not related to the intervention (unsystematic and systematic measurement error). bCBT‐I, blended cognitive behavioural therapy for insomnia following acquired brain injury (ABI); DBAS, Dysfunctional Beliefs and Attitudes about Sleep scale; ISI, Insomnia Severity Scale; SRBQ, Sleep‐Related Behaviours Questionnaire; TAU, treatment as usual.

The schematic model in Figure 1 can be translated into a regression model to test whether change in insomnia from T1 to T2 is associated with the changes in beliefs and behaviours. As it is hypothesized that bCBT‐I changes the beliefs and behaviours more than TAU, intervention can statistically be seen as a moderator changing the relations between beliefs and insomnia and between behaviours and insomnia.

### 2.2 Participants

A total of 52 participants were included from four outpatient rehabilitation centres spread over the Netherlands (Heliomare Rehabilitation, Wijk aan Zee; Basalt, The Hague; Adelante, Hoensbroek; Reade, Amsterdam). Data were collected between 2018 and 2020. Participants had ABI, and were referred to rehabilitation for multiple cognitive, emotional or behavioural complaints. The inclusion criteria were: (a) diagnosed with stroke or TBI; (b) 18 years or older; (c) Insomnia Severity Index (ISI) score ≥ 10; (d) insomnia according to DSM‐5 criteria; (e) comprehension of Dutch language. Exclusion criteria were: (a) untreated sleep apnea; (b) current or expected treatment of fatigue or sleep; (c) unstable medication regimens or medication with the side‐effect of insomnia; (d) alcohol abuse (> 3 glasses a day for at least 21 days per month) or drug abuse; (e) major untreated or unstable medical or psychiatric comorbid condition.

## 3 VARIABLES, DATA SOURCES, MEASUREMENT AND CONCEPTUALIZATION

### 3.1 Outcome measure: Insomnia

Insomnia was measured with the ISI (Bastien et al., 2001). The ISI consists of seven items to measure experienced insomnia in the participants using a five‐point scale. Total scores range from 0 (no insomnia) to 28 (severe insomnia). A score equal to and over 10 indicates a clinical level of insomnia (Lancee et al., 2015; Lancee et al., 2016; Morin et al., 2011). Internal consistency is adequate (Cronbach's alpha = 0.74–0.78) (Bastien et al., 2001; Morin et al., 2011).

### 3.2 Intervention

The bCBT‐I was based on standard CBT‐I, adapted to ABI (content and the way information was conveyed was adjusted to ABI), and consists of six online CBT sessions given on a weekly basis and personalized feedback after each session, combined with two face‐to‐face sessions, and an online sleep diary app for daily registration on their sleep. Participants were allowed to follow standard rehabilitation care for various complaints other than sleep, which is also the TAU‐condition. A more detailed description of the intervention is described elsewhere (Ford et al., 2020; Ford et al., 2022).

### 3.3 Moderators: Sleep‐related beliefs and behaviours

Sleep‐related beliefs were measured with the *Dysfunctional Belief and Attitudes about Sleep* scale (DBAS) (Morin et al., 2007). The DBAS consists of 16 items ranging from 0 (strongly disagree) to 10 (strongly agree) using a 11‐point scale. The scores of the 16 items were averaged, a higher score reflects more sleep‐disruptive beliefs. A DBAS average score of over 3.8 is associated with clinically significant insomnia (Hiller et al., 2015), and discriminates between self‐defined good and poor sleepers. The internal consistency is adequate (Cronbach's alpha = 0.79) (Morin et al., 2007).

Sleep safety behaviours were measured with the *Sleep‐Related Behaviours Questionnaire* (SRBQ) (Ree & Harvey, 2004). The SRBQ consists of 32 items to measure the extent to which safety behaviours are used to cope with sleep problems and tiredness on a five‐point scale. The total score ranges from 0 (no safety behaviours) to 128 (severe safety behaviours), and it discriminates between normal sleepers and people with insomnia (Ree & Harvey, 2004). The items of the SRBQ were developed based on the strategies that participants used in order to prevent feared sleep‐related outcomes (the dysfunctional beliefs of the DBAS) from occurring, and rated by clinical experts as “safety behaviours”. The internal consistency is sufficient (Cronbach's alpha = 0.83).

### 3.4 ABI‐adjusted moderators: ABI‐adapted sleep‐related beliefs and behaviours

Experts in our previous study agreed that not all items of the DBAS and SRBQ reflected dysfunctional beliefs and behaviours in the population of people with ABI. Only the items that seven (out of 10) or more ABI‐experts rated as dysfunctional were included in the ABI‐adjusted versions of the questionnaires (Verkaik et al., 2023). The ABI‐Adapted DBAS (DBAS‐ABI) consisted of 14 items (items 1, 2, 3, 4, 6, 7, 8, 9, 10, 11, 12, 13, 14 and 15 of the original DBAS). The ABI‐Adapted SRBQ (SRBQ‐ABI) consisted of 18 items (items 3, 4, 5, 6, 8, 10, 11, 12, 14, 15, 18, 23, 24, 26, 28, 29, 30 and 32 of the original SRBQ).

Additionally we made a subset of the questionnaire items that: (a) were discarded in the ABI‐adapted questionnaires; and (b) that seven (out of 10) or more ABI‐experts rated “not a dysfunctional belief” or “not a safety behavior” (Verkaik et al., 2023). In the SRBQ‐“not‐dysfunctional” subset, we included items 1, 2, 9, 17, 27, 31 (of the original SRBQ). There were no items to include in a DBAS‐“not‐dysfunctional” subset.

### 3.5 Statistical analyses

Descriptive statistics were used to summarize participants' characteristics. Demographic variables and clinical characteristics were compared with independent *t*‐tests, chi‐square tests and the Mann–Whitney *U*‐test where appropriate. Difference scores between pre‐ and post‐treatment were calculated for both the independent variables DBAS and SRBQ, and for the dependent variable (ISI). New interaction variables were created by multiplying DBAS change score and the intervention variable bCBT‐I (dummy score = 1) versus TAU (dummy score = 0) and SRBQ change score and the intervention variable in order to model the moderation. Two separate linear regression models were performed. The first regression analysis evaluated the extent to which changes in insomnia severity (ISI) between pre‐ and post‐treatment (dependent variable) were associated with changes in sleep‐related beliefs (DBAS), the type of intervention and the interaction effect of DBAS and the intervention (independent variables). The second regression analysis evaluated this for changes in sleep‐related behaviours (SRBQ). Type 1 error rate of 0.05 was used. These regression analyses were performed for ABI‐adapted questionnaires (DBAS‐ABI and SRBQ‐ABI) and the SRBQ‐“not‐dysfunctional” subset as well. Data were analysed per‐protocol, as this excluded the participants that deviated from the treatment protocol by not returning post‐treatment measurements or dropping out. As this study is not aimed to evaluate treatment efficacy, we considered a per protocol analysis more suitable to evaluate the association between a change in sleep‐related beliefs and behaviours and improvement of insomnia following bCBT‐I.

Missing values on the ISI, DBAS and SRBQ items were imputed when the percentage of missingness within a participant on a questionnaire was less than 10% (Bennett, 2001), and were replaced by participant mean on the other items of the same questionnaire. Moderation models were fitted with and without cases marked as outliers. Data analysis was performed with SPSS 24 (IBM; Armonck, USA).

## 4 RESULTS

Of the 52 participants enrolled in the RCT, two dropped out. Another two participants failed to return the entire post‐treatment measurement and were excluded. The 48 remaining participants were included in analyses (29 female and 19 male). Mean age at baseline was 51.9 years (SD 11.5). Median time since injury was 17.5 months (interquartile range [IQR] = 29). Educational level was low (primary education and less than 2 years of low level secondary school, or less) for six participants, average (finished low or average level secondary education) for 15 participants, and high (finished high level secondary education or a university degree) for 27 participants (Verhage, 1964). A total of 31 participants had a stroke, 17 a TBI. Detailed characteristics of the participants are shown in Table 1.

**TABLE 1:** Demographic and clinical characteristics (*N* = 48)

Characteristics	bCBT‐I (*n* = 24)	TAU (*n* = 24)	Total (*n* = 48)	*p*
Age at T1, years mean (SD)	51.9 (10.7)	51.9 (12.4)	51.9 (11.5)	0.99
Sex, *n* female (male)	13 (11)	16 (8)	29 (19)	0.39
Educational level^a^				0.32
Low (1–3), *n* (%)	2 (8)	4 (17)	6 (13)	
Average (4–5), *n* (%)	7 (30)	8 (33)	15 (31)	
High (6–7), *n* (%)	15 (62)	12 (50)	27 (56)	
Injury type				0.89
Stroke, *n* (%)	17 (71)	14 (58)	31 (65)	
TBI, *n* (%)	7 (29)	10 (42)	17 (35)	
TBI, mild	3	4	7	
TBI, moderate	3	5	8	
TBI, severe	1	1	2	
Time since injury, months mean (SD)	64 (131)	33 (50)	49 (99)	0.29
ISI				
Score at T1 mean (SD)	16.3 (7.3)	17.8 (4.4)	17.6 (4.1)	0.76
Score at T2 mean (SD)	11.3 (5.8)	15.8 (4.6)	13.5 (5.7)	< 0.01*
Delta (T1–T2) mean (SD)	6.1 (5.3)	2.1 (4.4)	4.1 (5.1)	< 0.01*
DBAS				
Score at T1 mean (SD)	4.9 (1.4)	5.4 (1.4)	5.2 (1.4)	0.23
Score at T2 mean (SD)	3.8 (1.4)	5.1 (1.5)	4.4 (1.6)	< 0.01*
Delta (T1–T2) mean (SD)	1.1 (1.0)	0.3 (1.1)	0.7 (1.1)	0.01*
SRBQ				
Score at T1 mean (SD)	46.5 (16.5)	47.4 (18.9)	47.0 (17.6)	0.86
Score at T2 mean (SD)	39.3 (14.7)	46.7 (18.1)	42.9 (16.7)	0.13
Delta (T1–T2) mean (SD)	8.3 (11.7)	1.2 (16.5)	4.7 (14.6)	0.10
HADS score at T1 mean (SD)	16.3 (7.3)	17.0 (7.2)	16.7 (7.2)	0.72

Abbreviations: bCBT‐I, blended cognitive behavioural therapy for insomnia; DBAS, Dysfunctional Beliefs and Attitudes about Sleep scale; HADS, Hospital Anxiety and Depression Scale; ISI, Insomnia Severity Index; SRBQ, Sleep‐Related Behaviours Questionnaire; TAU, treatment as usual; TBI, traumatic brain injury.

^a^ Education is based on Verhage.

* *p* < 0.05.

One pre‐treatment questionnaire was not returned, one post‐treatment questionnaire was not returned, one post‐treatment questionnaire had 43% missing values and was therefore not included in the analyses. Of all 285 (288 – 3) included questionnaires (*n* = 48), 15 out of 5209 values (0.29%) were missing and imputed. Two SRBQ values were considered an outlier, one was > 4SD below the mean and after visual analysis of the histogram, and one was > 2SD above the mean and turned out to be an influential data point. Both were excluded from analyses, but to get a complete picture all regressions were also fitted with the cases marked as an outlier and shown in the Results section. Based on detailed evaluation, there was no reason to assume that any assumption of a regression analysis was violated.

### 4.1 Sleep‐related beliefs

**FIGURE 2:**
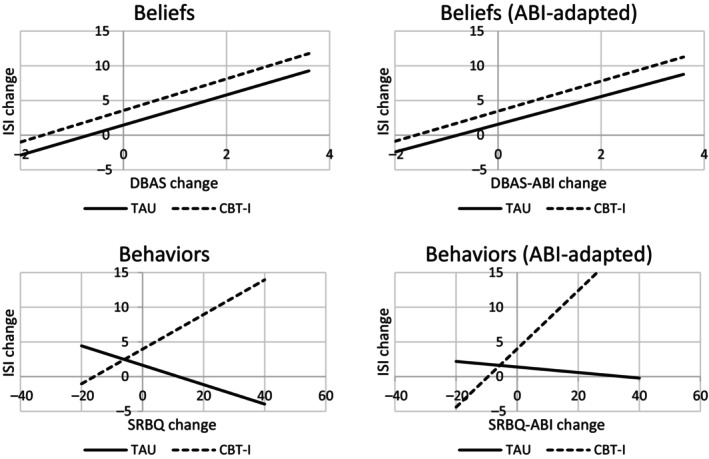
Regression interaction and main effects between Insomnia Severity Index (ISI), Dysfunctional Beliefs and Attitudes about Sleep scale (DBAS), Sleep‐Related Behaviours Questionnaire (SRBQ), and acquired brain injury (ABI) adapted versions, for treatment as usual (TAU) and blended cognitive behavioural therapy for insomnia (bCBT‐I).

At post‐treatment, there was a significant improvement on the DBAS following bCBT‐I, compared with TAU (*p* < 0.01; Table 1). The regression model to predict changes in insomnia by changes in DBAS, the type of the intervention (bCBT‐I versus TAU) and the interaction between DBAS change and intervention was significant, the explained variance of the total model was 35% (*F*_3,43_ = 7.74, *p* < 0.01). The regression weight for the DBAS change score was significant (*B* = 2.17, SE = 0.84, *p* = 0.01), indicating that positive change in DBAS led to positive change in insomnia score (Table 2, model 1). The type of intervention (*B* = 2.10, SE = 1.6, *p* = 0.20) and the interaction effect (*B* = 0.11, SE = 1.24, *p* = 0.93) were not significant, indicating that changes in DBAS lead to significant changes in insomnia, and this effect did not differ for participants in the bCBT‐I and TAU (see Figure 2 for the main effect of sleep‐related belief change scores in both bCBT‐I and TAU).

**TABLE 2:** Regression weights.

Coefficients^a^								
Model		Unstandardized coefficients		Standardized coefficients	*t*	Sig.	95% CI for *B*	
		B	SE	Beta			Lower	Upper
1 “Beliefs”	(Constant)	1.46	0.92		1.59	0.12	−0.39	3.32
	ΔDBAS	2.17	0.84	0.47	2.60	0.01*	0.48	3.85
	Type of intervention	2.10	1.62	0.21	1.30	0.20	−1.67	5.37
	Interaction	0.11	1.24	0.02	0.09	0.93	−2.39	2.60
2 “Behaviours”	(Constant)	1.64	0.97		1.69	0.10	−0.32	3.60
	ΔSRBQ	−0.14	0.11	−0.28	−1.22	0.23	−0.36	0.09
	Type of intervention	2.32	1.45	0.23	1.60	0.12	−0.61	5.26
	Interaction	0.39	0.13	0.70	2.87	< 0.01*	0.11	0.66
3 “Beliefs” ABI‐adapted	(Constant)	1.57	0.93		1.68	0.10	−0.31	3.44
	ΔDBAS‐ABI	2.00	0.80	0.44	2.49	0.02*	0.38	3.62
	Type of intervention	1.89	1.78	0.19	1.07	0.29	−1.69	5.48
	Interaction	0.17	1.30	0.02	0.13	0.90	−2.44	2.78
4 “Behaviours” ABI‐adapted	(Constant)	1.39	0.98		1.42	0.16	−0.59	3.37
	ΔSRBQ‐ABI	−0.04	0.19	−0.05	−0.22	0.83	−0.42	0.34
	Type of intervention	2.61	1.43	0.25	1.83	0.08	−0.27	5.49
	Interaction	0.46	0.22	0.54	2.07	< 0.05*	0.01	0.91

Abbreviations: ABI, acquired brain injury (adapted questionnaires); CI, confidence interval; ΔDBAS, Dysfunctional Beliefs and Attitudes about Sleep scale change scores; ΔSRBQ, Sleep‐Related Behaviours Questionnaire change scores.

^a^ Dependent variable is ISI change score (T1–T2).

* *p* < 0.05.

### 4.2 Sleep‐related behaviours

At post‐treatment, there was no significant difference in improvement on the SRBQ between participants in the sample following bCBT‐I compared with TAU (*p* = 0.13; Table 1). The regression model to predict changes in insomnia by changes in SRBQ, the type of intervention (bCBT‐I versus TAU) and the interaction between SRBQ change scores and intervention was significant, the explained variance of the total model was 40% (*F*_3,39_ = 8.65, *p* < 0.001). The regression weights for SRBQ change score (*B* = −0.14, SE = 0.11, *p* = 0.23) and intervention (*B* = 2.32, SE = 1.45, *p* = 0.12) were not significant (Table 2, model 2), but the interaction effect was (*B* = 0.39, SE = 0.13, *p* < 0.01). People's change in SRBQ scores were positively related to change in ISI scores in the bCBT‐I intervention, while there was no relationship between change in SRBQ scores and change in ISI scores for people in the TAU. The results of the regression analysis including the outliers were similar (*R* ^2^ = 0.34, *F*_3,41_ = 7.00, *p* = 0.001), individual effects of change in sleep‐related behaviours (*B* = −0.01, SE = 0.06, *p* = 0.85), intervention (*B* = 2.15, SE = 1.45, *p* = 0.15) and interaction (*B* = 0.26, SE = 0.01, *p* = 0.01; see Figure 2 for the interaction effect between sleep‐related behaviours change scores intervention).

### 4.3 Sleep‐related beliefs, ABI‐adaptation

The regression model to predict changes in insomnia by changes in DBAS‐ABI, the group (bCBT‐I versus TAU) and the interaction between DBAS‐ABI and group was significant, the explained variance of the total model was 32% (*F*_3,43_ = 6.87, *p* < 0.01). The regression weight for DBAS‐ABI change score was significant (*B* = 2.00, SE = 0.80, *p* = 0.02) (Table 2, model 3). The type of intervention (*B* = 1.89, SE = 1.78, *p* = 0.29) and the interaction effect (*B* = 0.17, SE = 1.30, *p* = 0.90) were not significant. The results indicated that positive changes in DBAS‐ABI lead to significant positive changes in insomnia, and this effect was similar for participants in the bCBT‐I and TAU (see Figure 2 for the main effect of ABI‐adapted sleep‐related belief change scores in both bCBT‐I and TAU).

### 4.4 Sleep‐related behaviours, ABI‐adaptation

The regression model to predict changes in insomnia by changes in SRBQ‐ABI, the type of intervention (bCBT‐I versus TAU) and the interaction between SRBQ‐ABI and intervention was significant, the explained variance of the total model was 41% (*F*_3,39_ = 8.91, *p* < 0.001). The regression weights for SRBQ‐ABI change score (*B* = −0.04, SE = 0.19, *p* = 0.83) and type of intervention (*B* = 2.61, SE = 1.43, *p* = 0.08) were not significant (Table 2, model 4), but the interaction effect was (*B* = 0.46, SE = 0.22, *p* < 0.05). Participants in the bCBT‐I intervention with larger changes in SRBQ‐ABI had significantly larger changes in insomnia scores compared with participants in TAU. The results of the regression analysis including the outliers were similar (*R* ^2^ = 0.37, *F*_3,41_ = 8.11, *p* < 0.001), individual effects of change in sleep‐related behaviours (*B* = 0.06, SE = 0.09, *p* = 0.54), intervention (*B* = 2.30, SE = 1.39, *p* = 0.11) and interaction (*B* = 0.36, SE = 0.15, *p* = 0.02).

See Figure 2 for the interaction effect between ABI‐adapted sleep‐related behaviours change scores and intervention.

### 4.5 Sleep‐related behaviours, “not‐dysfunctional” subset

The “not‐dysfunctional” subset of the SRBQ contains the items that were rated by ABI experts as “not dysfunctional” for people with ABI. The regression model to explore the changes in insomnia by changes in SRBQ‐“not‐dysfunctional” subset, the intervention (bCBT‐I versus TAU) and the interaction between SRBQ‐“not‐dysfunctional” and intervention was significant, the explained variance of the total model was 25% (*F*_3,39_ = 4.41, *p* < 0.01). The regression weight for intervention was significant (*B* = 4.19, SE = 1.47, *p* < 0.01). The regression weights for SRBQ‐“not‐dysfunctional” change score (*B* = –0.37, SE = 0.40, *p* = 0.35) and the interaction (*B* = 0.69, SE = 0.48, *p* = 0.16) were not significant. The results indicate that there is a significant difference between the type of intervention: bCBT‐I participants improved more in their insomnia severity than TAU participants. There is no main effect of the SRBQ‐“not‐dysfunctional” items on the level of the ISI and there is no significant interaction effect. In other words, the type of intervention was positively related to change in ISI scores, but changes in the SRBQ‐“not‐dysfunctional” subset did not lead to changes in insomnia. The results of the regression analysis including the outliers were similar (*R* ^2^ = 0.22, *F*_3,41_ = 3.75, *p* < 0.02), individual effects of change in sleep‐related behaviours “not‐dysfunctional” set (*B* = −0.24, SE = 0.27, *p* = 0.39), intervention (*B* = 3.94, SE = 1.43, *p* < 0.01) and interaction (*B* = 0.55, SE = 0.38, *p* = 0.16).

## 5 DISCUSSION

This study focused on whether decreases in insomnia severity are related to decreases in sleep‐related beliefs and behaviours, and whether they were influenced by bCBT‐I in patients with ABI. In line with our hypothesis, it was found that the bCBT‐I group improved significantly on dysfunctional beliefs (DBAS; *p* < 0.01) compared with the TAU group. Regression analysis additionally showed a positive effect for changes in beliefs (*p* = 0.02) on changes in insomnia severity. Improvement in dysfunctional beliefs was associated with improvement in insomnia severity after treatment, this was irrespective of treatment group (bCBT‐I or TAU). Also in line with our hypothesis, it was found that improvement in dysfunctional sleep‐related behaviours resulted in improvement in insomnia severity, but only in the bCBT‐I group and not in TAU (*p* < 0.01). So only in the bCBT‐I group is insomnia positively affected by improvement in behaviours (SRBQ).

Comparable results for the regression analyses with the ABI‐adapted questionnaires were found. Contrary to our expectation, there was no stronger association between bCBT‐I efficacy and sleep‐related beliefs and behaviours. It is noteworthy however that insomnia improvement following treatment could be predicted comparably with less items, including 14 out of the 16 original DBAS items, and 18 of the original 32 SRBQ items. Additionally, the items that were considered “not‐dysfunctional” did not lead to changes in insomnia. With caution, this strengthens the hypothesis that these items may indeed be not‐dysfunctional in the ABI population.

The results imply that especially changes in dysfunctional behaviours are involved in the change in insomnia following bCBT‐I in an ABI population and should be given attention in treatment. This is in line with findings in the general population, behavioural techniques are considered as the most active ingredients of the CBT‐I (Riemann et al., 2023), and that behavioural changes, such as adjusting time in bed and sleep schedule regularity, are associated to insomnia improvement (Parsons et al., 2021). Further investigation to identify the specific type of sleep‐related behaviours that affect insomnia in an ABI population is warranted.

Future research could clarify which specific beliefs and behaviours can be considered dysfunctional, and which can be considered not‐dysfunctional coping with the consequences of ABI. The questionnaire to assess sleep‐related behaviours (SRBQ) is developed based on strategies that people with insomnia use. Our study suggests that these strategies may be different for people with ABI, as almost half of the items seem not relevant in our study population. It could be useful to construct a new questionnaire or adapted version for people with ABI, using a similar systematic process as has been used in the constructing of the original questionnaire (Ree & Harvey, 2004). For a better understanding of the cause–effect or temporal relation between beliefs, behaviours and insomnia improvement, frequently repeated measurements during treatment, in addition to the pre‐ and post‐treatment measurements, could be helpful.

### 5.1 Strengths and limitations

This study has several strengths. To our best knowledge this is the first study to examine the association between change in sleep‐related beliefs and behaviours and post‐treatment improvement in insomnia in the ABI population. Improvements in sleep‐related beliefs and behaviours following bCBT‐I is not only evaluated, but also related to actual changes in insomnia and treatment efficacy in an ABI population. The results were in line with findings of both online CBT‐I (Lancee et al., 2015) and face‐to‐face CBT‐I (Parsons et al., 2021) in the general population, suggesting that changing dysfunctional beliefs and especially changing behaviours plays an important role in treatment outcome. Analyses of the ABI‐adapted questionnaires strengthen the importance to determine which items reflect dysfunctional and which items reflect not‐dysfunctional beliefs and behaviours.

Some limitations of the current study should be acknowledged. Firstly, both the DBAS and SRBQ were developed for the otherwise healthy population. To anticipate for this, we also used adapted versions for the ABI population, but these versions included less, but the same items. No new possibly additional dysfunctional beliefs and behaviours that may be specific for people with ABI were added. However, our results indicate that the concepts of dysfunctional beliefs and behaviours are also relevant to insomnia in an ABI population. Secondly, a psychometric limitation is that we focused mainly on difference scores both as dependent and independent variables. A property of difference scores in general is that those scores contain the measurement errors of both administrations. As measurement error is per definition not related to anything else, this may have led to lower correlations between dependent and independent variables than the true association. Thirdly, the relatively small sample size is a limitation for several reasons. Ideally both changes in beliefs and changes in behaviour should be added in one regression model simultaneously in order to evaluate the effect of the intervention on changes in sleep, but this was not possible due to small sample size. Also, to strengthen the conclusion that CBT‐I is moderated by improvement in behaviours requires a moderation analysis with a larger sample size. A larger sample size is also needed to evaluate the impact of type and severity of the ABI and its neuropsychological consequences on the results, as there may be a large heterogeneity in recovery and outcome. Lastly, it is important to stress that this study focused solely on beliefs and behaviours as contributing factors to treatment efficacy, and found that 40% of variance in insomnia severity at post‐treatment could be explained by these factors. Using a broader approach including other factors that affect sleep following brain injury could be valuable for a more complete understanding of insomnia treatment in people with ABI. Based on the model of Ouellet et al. (2015), these could include pre‐injury, peri‐injury, acute and post‐acute factors (Ouellet et al., 2015) and, based on the general population, other mechanisms that may perpetuate insomnia are also worth to be investigated in an ABI population, such as cognitive, behavioural and hyperarousal factors (Schwartz & Carney, 2012).

## 6 CONCLUSION

This study contributes to a better understanding of how sleep‐related beliefs and behaviours are interrelated to insomnia following ABI. Improvement on insomnia severity is related to improvement in dysfunctional beliefs and behaviours, and bCBT‐I efficacy in this study is moderated by the improvement in behaviours, but larger studies are needed to strengthen the conclusion that efficacy of CBT‐I is moderated by changing behaviour in general. Although this preliminary finding needs to be validated in future studies, the results suggest that a focus on these behaviours may enhance treatment efficacy, but also that caution is needed regarding the behaviours that may reflect adequate coping with the consequences of the ABI. For clinical purposes, questionnaires should be validated in the ABI population or used with caution.

## AUTHOR CONTRIBUTIONS

**Marthe E. Ford:** Conceptualization; data curation; formal analysis; investigation; methodology; project administration; writing – original draft; writing – review and editing. **Frank Verkaik:** Investigation; conceptualization; writing – original draft; data curation; formal analysis; project administration; methodology. **Samantha Bouwmeester:** Data curation; methodology; formal analysis; writing – original draft. **Gert J. Geurtsen:** Conceptualization; formal analysis; supervision; methodology; writing – original draft; writing – review and editing.

## FUNDING INFORMATION

The RCT that provided the data for this study was supported by the Dutch Brain Foundation (Hersenstichting: Grant no. DR2019‐003377). There was no role for the funders in study design, data collection, analysis, manuscript preparation or decision to publish. The study was performed at the Department of Psychology, Heliomare Rehabilitation, Wijk aan Zee, The Netherlands.

## CONFLICT OF INTEREST STATEMENT

No potential conflict of interest is reported by the authors.

## Data Availability

The data that support the findings of this study are available on request from the corresponding author. The data are not publicly available due to privacy or ethical restrictions.
